# Liraglutide Effectiveness in Type 2 Diabetes: Insights from a Real-World Cohort of Portuguese Patients

**DOI:** 10.3390/metabo12111121

**Published:** 2022-11-16

**Authors:** José Silva-Nunes, Edite Nascimento, Joana Louro, Jorge Dores, Teresa Laginha, Ana Gonçalves-Ferreira, Marta Alves, Selma B. Souto, Nelson Cunha, Elsa Pina, Rui Duarte, João Filipe Raposo

**Affiliations:** 1 Department of Endocrinology, Diabetes and Metabolism, Curry Cabral Hospital—Centro Hospitalar Universitário de Lisboa Central, 1050-099 Lisboa, Portugal; 2 NOVA Medical School, New University of Lisbon, 1169-056 Lisboa, Portugal; 3 Health and Technology Research Center (H&TRC), Escola Superior de Tecnologia da Saúde de Lisboa, 1990-096 Lisboa, Portugal; 4 Department of Internal Medicine, Centro Hospitalar Tondela-Viseu, 3504-509 Viseu, Portugal; 5 Department of Internal Medicine, Centro Hospitalar do Oeste, 2500-176 Caldas da Rainha, Portugal; 6 Department of Endocrinology, Diabetes and Metabolism, Centro Hospitalar Universitário do Porto, 4099-001 Porto, Portugal; 7 Diabetes Clinic, Associação Protetora dos Diabéticos de Portugal (APDP), 1250-189 Lisboa, Portugal; 8 Department of Endocrinology, Diabetes and Metabolism, Garcia de Orta Hospital, 2805-267 Almada, Portugal; 9 Department of Endocrinology, Diabetes and Metabolism, Hospital de Braga, 4710-243 Braga, Portugal; 10 Department of Endocrinology, Diabetes and Metabolism, Centro Hospitalar e Universitário de Coimbra, 3000-075 Coimbra, Portugal; 11 Department of Internal Medicine, Centro Hospitalar Universitário do Algarve, 8000-386 Faro, Portugal; 12 Portuguese Society of Diabetology (SPD), 1250-198 Lisboa, Portugal

**Keywords:** liraglutide, real-world evidence, type 2 diabetes, glycaemic control, anthropometric parameters, cardiovascular risk factors

## Abstract

Liraglutide is a long-acting glucagon-like peptide-1 receptor agonist prescribed to diabetic patients for glycaemic control. To understand the impact of liraglutide in the real-world setting, this study analysed its effects in a Portuguese cohort of Type 2 diabetes patients. This was an observational, multicentric, and retrospective study that included 191 liraglutide-treated patients with at least 12 months of treatment. Patients’ data were collected and analysed during a 24-month follow-up period. Overall, liraglutide treatment effectively reduced HbA1c levels from 8.3% to around 7.5%, after 6, 12, and 24 months (*p* < 0.001). In fact, 38.2%, 37.2%, and 44.8% of patients at 6, 12, and 24 months, respectively, experienced an HbA1c reduction of at least 1%. Moreover, a persistent reduction in anthropometric features was also observed, with 44.0%, 47.6%, and 54.4% of patients achieving a weight reduction of at least 3% at 6, 12, and 24 months, respectively. Finally, significant improvements were observed in the HDL-c and LDL-c levels. Our results demonstrate that liraglutide effectively promoted the reduction of HbA1c values during routine clinical practice, which was sustained throughout the study. In addition, there were significant improvements in anthropometric parameters and other cardiovascular risk factors.

## 1. Introduction

Despite the global effort put into diabetes mellitus (DM) research and patient education, this condition remains a worrisome public health issue. Intimately linked with the increasing prevalence of obesity, Type 2 DM (T2DM) accounts for approximately 90% of all DM cases and is a major cause of several micro- and macrovascular complications [1,2].

Upon food ingestion, the incretin glucagon-like peptide-1 (GLP-1) is released and stimulates insulin secretion while reducing glucagon production [3,4,5,6]. In addition, it decreases gastric motility, promotes satiety, may have a cardioprotective role besides glycaemic control (namely, by reducing blood pressure [BP] and post-prandial triglycerides [TG]/free fatty acid levels), and inhibits ß-cell apoptosis while inducing its proliferation [4,5]. However, endogenous GLP-1 is not suitable for long-term action, since its circulating form is rapidly degraded by dipeptidyl peptidase 4 (DPP-4).

The pharmaceutical industry has therefore invested in the development of a group of peptides that mimic GLP-1 function, the GLP-1 receptor agonists (GLP-1ra). They are resistant to DPP-4 degradation, allowing for supra-physiological GLP-1 equivalent concentrations and representing a suitable option for controlling hyperglycaemia when lifestyle modifications and metformin therapy are unable to do so [3,5]. Thus, GLP-1ra are currently recommended in clinical practice as a prognosis modifier for T2DM, even in the presence of good metabolic control.

Liraglutide is a long-acting GLP-1ra with 97% sequence homology with the native hormone [3,5]. Its efficacy and safety as a monotherapy or add-on treatment for adult T2DM patients have been demonstrated in an extensive clinical trial programme named LEAD (Liraglutide Effect and Action in Diabetes). Briefly, liraglutide effectively reduced glycated haemoglobin (HbA1c) and blood glucose levels, increased insulin levels, improved surrogate markers of ß-cells function, promoted weight loss, and reduced BP [3,7,8,9,10,11,12,13,14,15,16,17]. As for safety concerns, liraglutide was generally well tolerated, with reports of relatively frequent—but moderate and transient—gastrointestinal adverse events, namely, nausea and vomiting [3,7,8,9,10,11,12,13]. Major hypoglycaemic episodes were uncommon, likely due to liraglutide’s glucose-dependent mechanism of action [3,7,8,9,10,11,12,13]. Additional clinical trials involving T2DM patients with a high cardiovascular risk have demonstrated the cardioprotective potential of liraglutide, which decreased the risk of cardiovascular events and improved several cardiometabolic risk indices [18,19,20]. Finally, liraglutide was also shown to be associated with lower rates of the development and progression of diabetic kidney disease, as compared to placebo [21].

The added value of liraglutide in the pharmacological approach to T2DM has been firmly established by several randomised clinical trials. However, data from the trials are seldom replicated during standard clinical practice, mainly due to the strict inclusion criteria, the high patient compliance, and the close monitoring routine that characterises this type of approach. For instance, adjusting the eligibility criteria of the LEAD 1–5 trials to the Danish population revealed that only 26.9% of the 9251 patients prescribed liraglutide would have been eligible for these trials [22].

Observational studies and post-marketing analyses are, therefore, of utmost importance to ascertain the impact of liraglutide in the real world, as well as to unveil its long-term effects. With that in mind, and with the goal of contributing to filling the knowledge gap between randomised clinical trials and real-world practice, we have analysed the effects of liraglutide in a Portuguese cohort of T2DM patients in regard to glycaemic control, changes in anthropometric parameters, and variations in other cardiovascular risk factors.

## 2. Materials and Methods

### 2.1. Design Overview, Setting, and Participants

This was an observational, retrospective, and multicentric study designed to evaluate the effectiveness of liraglutide in a population of Portuguese T2DM patients in ambulatory care. Patients were recruited by medical investigators from 10 outpatient diabetes clinics previously selected by the Portuguese Society of Diabetology (Sociedade Portuguesa de Diabetologia, SPD). Adult T2DM patients who were prescribed liraglutide, either alone or in combination therapy, were invited to participate in the study. Inclusion criteria were (i) at least 12 months of liraglutide treatment at enrolment and (ii) treatment according to Victoza^®^ Summary of Product Characteristics (SmPC). This study was approved by the Ethics Committee (Comissão de Ética para a Saúde, approval number: 460/2017). All participants signed a written informed consent form before study enrolment and data collection.

### 2.2. Assessments and Endpoints

The primary efficacy outcome was the percentage of patients who achieved an HbA1c reduction of at least 1% at a 12-month follow-up. The secondary efficacy outcomes included: the percentage of patients who achieved an HbA1c reduction of at least 1% at 6- and 24-month follow-ups; the percentage of patients who achieved an HbA1c goal of ≤6.5%, ≤7.0%, or ≤7.5%, at 6-, 12-, and 24-month follow-ups; the percentage of patients who had a weight reduction of at least 3% or 5% at 6-, 12-, and 24-month follow-ups; the percentage of patients who simultaneously achieved an HbA1c reduction of at least 1.0% and a weight reduction of at least 3.0% at 6-, 12-, and 24-month follow-ups; and the changes in HbA1c, body weight, body fat mass, waist circumference, lipid profile, and systolic/diastolic BP between the baseline and 6-, 12-, and 24-month follow-ups.

### 2.3. Data Collection

Data were retrospectively collected from an SPD-managed electronic platform containing patients’ case report forms. Data collection was performed by an independent contract research organisation (W4Research).

At study enrolment, demographic (sex and gender) and clinical data (disease duration and concomitant treatment to liraglutide), HbA1c values, anthropometric parameters (weight, BMI, body fat mass, and waist circumference), and cardiovascular risk markers (BP, high-density lipoprotein cholesterol [HDL-c], low-density lipoprotein cholesterol [LDL-c], and triglycerides [TG] levels) were collected. In the 6-, 12-, and 24-month follow-up visits, all of the above-mentioned variables, except for demographics, were collected.

### 2.4. Statistical Analysis

Continuous variables were described as the median and 25th and 75th percentiles (P25 and P75) and were analysed using linear (or additive) generalised mixed-effects models with associated identity function. Categorical variables were described with absolute and relative frequencies and were analysed using linear (or additive) generalised mixed-effects models with associated logit function. The Bonferroni correction method was used to adjust the significance level in the presence of multiple comparisons.

Univariate and multivariate logistic regression models were adjusted to determine predictors for a reduction of at least 1% in the HbA1c levels. Estimates of the coefficients and their associated standard error for each independent variable included in the final model were presented, as well as the respective *p*-values, the adjusted odds ratio estimates, and the respective 95% confidence intervals.

A significance level of 0.05 was used. Data were compiled and analysed using the R software.

## 3. Results

### 3.1. Cohort Characterisation

This study cohort included 191 adult T2DM patients, whose baseline characteristics are depicted in Table 1. Most patients (116, 60.7%) were female, with an overall median (interquartile range [IQR]) HbA1c level of 8.3% (7.3; 9.2). The cohort’s median age and disease duration were 59.0 (52.0; 65.0) and 12.0 (6.0; 17.0) years, respectively.

Concerning anthropometric parameters, patients weighed a median of 95.0 Kg (84.1; 105.2) and had a 41.0% (32.0; 46.3) body fat mass and a waist diameter of 113.0 cm (106.0; 123.0). The median systolic and diastolic BP were 140.0 mmHg (128.0; 156.0) and 82.0 mmHg (72.0; 90.0), respectively. The median total cholesterol was 162.0 mg/dL (139.0; 196.0), of which 42.0 mg/dL (36.0; 50.0) was HDL-c and 95.0 mg/dL (76.0; 125.0) was LDL-c. The TG median level was 142.0 mg/dL (106.3; 191.8).

Concomitantly with liraglutide, patients were taking other anti-diabetic medications, with metformin being the most common (prescribed to 87.4% of patients), followed by DPP-4 inhibitors (DPP4i) (63.4%), insulin (56.5%), sulphonylureas (22.5%), acarbose (9.9%), dapagliflozin (3.7%), and pioglitazone (3.1%) (Table S1). Additionally, 83.2% of patients were on anti-hypertensives, and 79.1% were on lipid-lowering drugs. The frequency of use of each specific medication at baseline and at each follow-up visit is listed in Table S1.

### 3.2. Clinical Effectiveness

#### 3.2.1. Glycaemic Control

**Figure 1:**
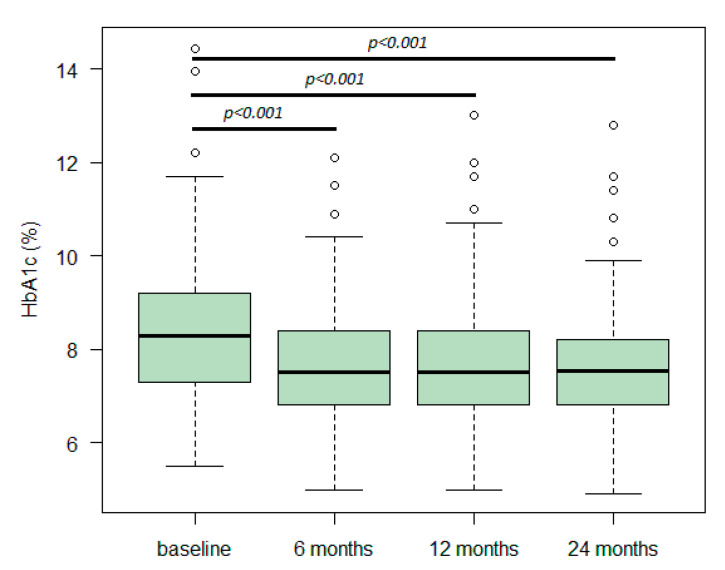
HbA1c (%) variation over time. HbA1c variation from baseline to 24 months. Boxes indicate the median and the P25/P75 percentiles, whiskers indicate the range of values, and dots correspond to outliers. Statistical analysis was performed using a linear generalised mixed-effects model with an associated identity function.

The HbA1c variation over time is illustrated in Figure 1. HbA1c median values were 7.5% after 6 and 12 months of study initiation and 7.6% after 24 months, all significantly lower than the 8.3% registered at baseline (*p* < 0.001, for all timepoints).

In line with the primary outcome of the study, 12 months after study initiation, the percentage of patients who achieved an HbA1c reduction of at least 1% was 37.2%. In addition, 38.2% and 44.8% of patients showed a reduction of at least 1% in HbA1c at 6 and 24 months, respectively. Of the patients who completed the 24-month follow-up (*n* = 131), 21.4% had a reduction of at least 1% in their HbA1c at all time points.

A higher probability for a patient to achieve at least a 1% reduction in the HbA1c levels was associated with having a baseline HbA1c level > 7% (*p* = 0.001, OR = 28.420) or taking insulin (*p* = 0.006, OR = 2.483). On the other hand, taking metformin (*p* = 0.005, OR = 0.288) or taking acarbose (*p* = 0.047, OR = 0.220) were predictors for not achieving such a reduction in HbA1c levels (Table S2).

Multivariable logistic regression analysis confirmed that patients who had an HbA1c basal level > 7% were more likely to achieve an HbA1c level reduction of at least 1% (*p* = 0.001, OR = 27.691). On the other hand, the probability of such a reduction was highly decreased in patients taking metformin (*p* = 0.014, OR = 0.304) (Table S3).

The percentages of patients who achieved the HbA1c goals of ≤6.5%, ≤7.0%, or ≤7.5% at each follow-up visit were statistically significantly higher than those observed at baseline for that specific HbA1c goal (Table 2). By the end of the study, HbA1c values smaller than or equal to 6.5%, 7.0%, and 7.5% were observed in approximately 20%, 35%, and 50% of patients, respectively.

Regarding the frequency of patients taking anti-diabetics during the study period, it was higher with sulphonylureas (6 months: *p* = 0.001; 12 months: *p* = 0.010, 24 months: *p* = 0.026) and DPP4i (*p* = 0.000, for all time points) at baseline, when compared to the other time points, and with acarbose at baseline, when compared to the 6-month follow-up (*p* = 0.040) (Table S1). On the other hand, more patients were on dapagliflozin at the 24-month follow-up (*p* = 0.000) compared to baseline.

#### 3.2.2. Anthropometric Parameters

The variations in weight, body mass index (BMI), body fat mass, and waist circumference throughout the study are depicted in Table 3.

Body weight, BMI, and body fat mass were significantly reduced at all three time points when compared to baseline (body weight and BMI: *p* < 0.001 for all; body fat mass: *p* = 0.001, *p* < 0.001, and *p* = 0.002, at 6, 12, and 24 months, respectively). The reduction in waist circumference was only statistically different after 6 (*p* = 0.002) and 12 months (*p* = 0.001) of study initiation.

At the 6-month follow-up visit, the median weight variation was −2.7% (ranging from −15.2 to +13.4%), the median body fat variation was −2.7% (ranging from −35.0 to +13.8%), and the median waist diameter variation was −1.7% (ranging from −17.6 to +11.8%). At the 12-month visit, the median weight variation was −2.7% (ranging from −20.7 to +13.0%), the median body fat variation was −4.4% (ranging from −25.6 to +22.0%), and the median waist diameter variation was −1.8% (ranging from −18.4 to +15.9%). Finally, at the 24-month visit, the median weight variation was −3.5% (ranging from −37.2 to +16.4%), the median body fat variation was −5.7% (ranging from −35.9 to +15.0%), and the median waist diameter variation was 0.0% (ranging from −19.2 to +25.6%).

In terms of goals, a weight reduction of at least 3% was achieved by 44.0%, 47.6%, and 54.4% of all patients at 6, 12, and 24 months, respectively, whereas a weight reduction of at least 5% was achieved by 24.5%, 31.4%, and 37.5% of patients at the same time points (Table S4). Of note, 18.2% (at 6 months), 17.3% (at 12 months), and 22.6% (at 24 months) of patients simultaneously achieved a weight reduction of at least 3% and an HbA1c reduction of at least 1%.

#### 3.2.3. Cardiovascular Risk Markers

Table 4 depicts the variation in patients’ BP and lipid profile over time for those who did not change their BP-lowering or anti-dyslipidaemia medication throughout the study.

HDL-c and LDL-c levels were statistically significantly altered 24 months after study initiation. HDL-c levels increased (*p* = 0.038), whereas LDL-c levels decreased (*p* = 0.033). There were no significant differences in the variation in systolic and diastolic BP, total cholesterol, and TG levels.

As for the lipid profile, the median variations (P25; P75) between the 6-, 12-, and 24-month visits and baseline were as follows: total cholesterol = −4.0 (−23.0; 9.0), −3.0 (−22.0; 9.0), and −5.0 (−19.5; 9.0), respectively; LDL-c = −5.0 (−19.5; 10.0), −6.0 (−13.0; 7.0), and −7.0 (−21.5; 5.5), respectively; HDL-c = 0.0 (−3.3; 2.0), 0.0 (−4.0; 4.0), and 3.0 (−2.0; 6.0), respectively; and TG = 5.0 (−28.0; 11.0), −5.5 (−33.5; 20.8), and −10.0 (−34.8; 23.0), respectively.

Among those taking anti-dyslipidaemia medication, more patients were on statins at the 6-month follow-up compared with baseline (*p* = 0.010). No other differences were found in the percentage of patients treated with other types of anti-dyslipidaemia or anti-hypertensive drugs.

## 4. Discussion

We have retrospectively analysed the effectiveness of liraglutide in a population of 191 Portuguese T2DM patients from 10 outpatient clinical centres, in terms of glycaemic control, anthropometric parameters, and other cardiovascular risk markers. Most patients were female (60.7%), obese (median BMI: 35.6), and under anti-diabetic (98.4%), anti-hypertensive (83.2%), and/or anti-dyslipidaemia medications (79.0%). Such characteristics are in accordance with previous studies conducted in the Portuguese population with T2DM, which describe a higher prevalence of the disease among women [23,24], obesity as one of the most common comorbidities [23], and the frequent use of the aforementioned medication [24]. Overall, most analysed variables have shown a statistical and clinical improvement 6 months after the introduction of liraglutide therapy, which was sustained for up to 24 months.

Our results show that liraglutide treatment resulted in glycaemic control, as reflected by the significant decrease in HbA1c values at the 6-, 12-, and 24-month evaluations. Even though this reduction is in line with what was observed in the LEAD 3 trial and several real-world studies [13,15,16,25,26], others have reported an HbA1c reduction that is somewhat higher [8,9,10,11,12,26,27,28,29,30,31,32,33,34]. A similar pattern was detected in the percentage of patients who achieved the predetermined HbA1c goals of ≤6.5%, ≤7.0%, and ≤7.5%. Still, it is worth mentioning that 38.2% of all patients had an HbA1c reduction of at least 1% 12 months after study initiation, a value that increased to 44.8% at the 24-month follow-up visit.

Despite being modest, the reduction in the HbA1c levels suggests that most patients responded to liraglutide treatment. The main reason for this somehow modest yet statistically and clinically significant decrease in HbA1c may be related to the significant proportion of patients with HbA1c levels ≤7% (31.4%) at baseline. Such values were not expected to decrease by more than 1%, independently of the anti-diabetic medication introduced. In addition, even though the baseline HbA1c level was similar to that of previous studies, patients presented with a severe disease that lasted for a long time (12 years), with 56.5% of them already on insulin therapy at baseline. Another plausible reason could be related to the observed changes in the T2DM medication, such as the high frequency of patients (nearly two-thirds) who were under DPP4i at the time of study initiation and the reduction in the prescription of sulphonylureas. Of note, some patients who were previously on DPP4i incorrectly maintained this therapy after starting liraglutide.

As for the reduction of at least 1% in HbA1c levels, as expected, this was more likely to occur in patients with higher levels at baseline. The results showed that such a reduction was nearly 28 times more probable to occur in patients with an HbA1c basal level > 7% but less likely to occur in those taking metformin. The latter is an intriguing observation given that this medication was prescribed to more than 80% of patients during the 24-month study period.

We also noted a sustained improvement in the anthropometric parameters. Six months after treatment initiation, there was a decrease in median body weight (2.1 kg), body fat mass percentage (2.0%), and waist circumference (1.0 cm). These alterations were significant when compared to the baseline and similar to those at 12 and 24 months. Concerning weight reduction, we observed slightly higher values than reported in most LEAD trials [8,9,10,11,12,13]. Nevertheless, real-world studies report a wide range of weight reduction values among liraglutide users [15,17,26,27,28,29,30,32,33,34,35], a variation that likely reflects the baseline population characteristics, the concomitant medication, the follow-up time, the fact that some protocols focus on overweight or obese patients, and the dietary patterns of the study population. With respect to waist circumference, the reduction reported here is within the range of what has been previously published [8,28,34]. Although seemingly small, this reduction is clinically relevant given the importance of this parameter with regard to cardiometabolic risk [36].

Finally, we have found a persistent improvement in some of the cardiovascular risk factors analysed, such as the increase in HDL-c and the decrease in LDL-c levels, and an overall modification in the lipid profile, which was most consistent with the results published by others [9,12,29,34]. Still, in this cohort, the reduction in LDL-c was smaller than those identified by Buse et al. and Rondinelli et al. [12,28], and the TG values did not significantly change, whereas the LEAD trials 4 and 6 and one observational study have found them to decrease [9,12,34]. Concerning patients who did not change their BP-lowering medication, systolic and diastolic BP did not suffer significant alterations, in contrast to what was found in most LEAD randomised clinical trials and some observational studies [8,10,11,12,13,28,29,31,34]. These disparities are likely related to the patients’ baseline cardiovascular risk, follow-up time, and concomitant therapies.

In the future, it would be interesting to achieve a follow-up of this cohort to evaluate liraglutide’s effectiveness for a longer period under real-world conditions. Additionally, it would be beneficial to perform a more thorough characterisation of the patients’ sociodemographic and clinical variables, as well as the collection of adverse events.

Our study has some key strengths worth mentioning. Its multicentric nature accounts for a good representation of the Portuguese T2DM outpatient population in a real-world setting, and its relatively long follow-up period allows for the analysis of liraglutide effects from a long-term perspective. However, there are also a few limitations that should be acknowledged. The main one is inherent to all observational studies and is related to the non-controlled and non-randomised design of the study, which allows for the presence of selection bias and uncontrolled variables affecting the results. In addition, due to its retrospective nature, several parameters, such as laboratory data, could not be evaluated.

## 5. Conclusions

In conclusion, we have shown that the use of liraglutide in routine clinical practice was effective in terms of metabolic control, as it also promoted weight loss, waist circumference reduction, and lipid profile improvement. Importantly, most of these effects were visible 6 months after study initiation and were sustained for at least two years. Our observations add to the body of evidence supporting the role of liraglutide as an attractive option for the management of T2DM, especially in patients who are overweight and/or have multiple cardiovascular risk factors.

## Figures and Tables

**Table 1:** Patient demographics and baseline characteristics.

		Median (IQR) ^1^
Sex, *n* (%)	female	116 (60.7%)
	male	75 (39.3%)
Age (years) ^2^		59.0 (52.0; 65.0)
Disease duration (years) ^2^		12.0 (6.0; 17.0)
HbA1c (%)		8.3 (7.3, 9.2)
Body weight (Kg)		95.0 (84.1, 105.2)
BMI (Kg/m^2^)		35.6 (31.7; 39.5)
Body fat mass (%)		41.0 (32.0, 46.0)
Waist diameter (cm)		113.0 (106.0, 123.0)
Systolic BP (mmHg)		140.0 (128.0, 156.0)
Diastolic BP (mmHg)		82.0 (72.0–90.0)
Total cholesterol (mg/dL)		162.0 (139.0–196.0)
HDL-c (mg/dL)		42.0 (36.0–50.0)
LDL-c (mg/dL)		95.0 (76.0–125.0)
TG (mg/dL)		142.0 (106.3–191.8)

^1^ except when noticed; ^2^ at the onset of liraglutide therapy. BMI: Body mass index; HbA1c: glycated haemoglobin; BP: blood pressure; HDL-c: high-density lipoprotein cholesterol; LDL-c: low-density lipoprotein cholesterol; TG: triglycerides.

**Table 2:** Number of patients (*n*/%) who achieved different HbA1c goals over time.

	Baseline (*n* = 191)	6 Months (*n* = 182)	12 Months (*n* = 191)	24 Months (*n* = 134)
HbA1c ≤ 6.5%	18 (9.4%)	33 (18.1%)	35 (18.3%)	27 (20.1%)
		*p* = 0.001	*p* < 0.001	*p* = 0.001
HbA1c ≤ 7.0%	42 (22.0%)	64 (35.2%)	69 (36.1%)	48 (35.8%)
		*p* < 0.001	*p* < 0.001	*p* = 0.010
HbA1c ≤ 7.5%	61 (31.9%)	93 (51.1%)	100 (52.4%)	67 (50.0%)
		*p* < 0.001	*p* < 0.001	*p* = 0.003

Statistical analysis was performed using a linear generalised mixed-effects model with an associated identity function. The *p*-values correspond to the comparison of frequencies between the baseline and each time point. HbA1c: glycated haemoglobin.

**Table 3:** Variation in anthropometric characteristics over time (median [IQR]).

	Baseline	6 Months	12 Months	24 Months
Weight (Kg)	95.0 (84.1; 105.2)	92.8 (80.7; 103.5)	92.0 (80.0; 103.7)	92.5 (81.3; 101.3)
	*n* = 191	*p* < 0.001, *n* = 184	*p* < 0.001, *n* = 191	*p* < 0.001, *n* = 136
BMI (Kg/m^2^)	35.6 (31.7; 39.5)	34.4 (30.8; 39.0)	34.2 (30.4; 38.1)	34.0 (30.8; 38.7)
	*n* = 182	*p* < 0.001, *n* = 176	*p* < 0.001; *n* = 182	*p* < 0.001; *n* = 133
Body fat mass (%)	41.0 (32.0; 46.0)	39.0 (32.0; 46.3)	39.0 (31.0; 45.0)	35.0 (29.0; 43.0)
	*n* = 59	*p* = 0.010, *n* = 58	*p* < 0.001, *n* = 55	*p* = 0.002, *n* = 36
Waist circumference (cm)	113.0 (106.0; 123.0)	112.0 (103.0; 118.5)	111.0 (104.5; 119.0)	111.0 (104.0; 118.0)
	*n* = 111	*p* = 0.002, *n* = 101	*p* = 0.001, *n* = 97	*p* = 0.513, *n* = 67

Statistical analysis was performed using a linear generalised mixed-effects model with an associated identity function. The *p*-values correspond to the comparison of each anthropometric characteristic between the baseline and each time point. BMI: body mass index.

**Table 4:** Variation in cardiovascular risk indicators over time in patients who did not change their BP/lipid-lowering medications (median [IQR]).

	Baseline	6 Months	12 Months	24 Months
Systolic BP (mmHg)	137.0 (127.0–150.0)	136.0 (125.0–148.0)	138.5 (125.0–155.0)	136.0 (127.0–149.5)
	*n* = 115	*p* = 0.175, *n* = 107	*p* = 0.779, *n* = 114	*p* = 0.467, *n* = 81
Diastolic BP (mmHg)	81.0 (71.0–88.0)	80.0 (74.0–86.0)	80.0 (74.0–89.0)	79.0 (71.5–87.5)
	*n* = 115	*p* = 0.083, *n* = 107	*p* = 0.629, *n* = 115	*p* = 0.201, *n* = 81
Total cholesterol (mg/dL)	154.0 (135.0–182.0)	148.5 (129.8–174.0)	155.0 (135.5–172.5)	154.0 (129.3–170.3)
	*n* = 75	*p* = 0.122, *n* = 68	*p* = 0.221, *n* = 73	*p* = 0.091, *n* = 56
HDL-c (mg/dL)	42.0 (36.0–48.0)	42.0 (34.0–50.0)	40.0 (34.0–50.0)	43.0 (36.5–52.5)
	*n* = 76	*p* = 0.068, *n* = 68	*p* = 0.983, *n* = 70	*p* = 0.038, *n* = 57
LDL-c (mg/dL)	92.0 (74.0–115.0)	86.0 (62.0–108.5)	86.0 (67.3–110.8)	85.0 (63.0–104.0)
	*n* = 75	*p* = 0.242, *n* = 73	*p* = 0.100, *n* = 72	*p* = 0.033, *n* = 59
TG (mg/dL)	137.0 (99.0–173.5)	122.0 (87.3–186.0)	125.0 (99.0–182.0)	137.0 (86.0–201.0)
	*n* = 77	*p* = 0.062, *n* = 72	*p* = 0.061, *n* = 75	*p* = 0.585, *n* = 59

Statistical analysis was performed using a linear generalised mixed-effects model with an associated identity function. The *p*-values correspond to the comparison of each cardiovascular risk factor between baseline and each time point. BP: blood pressure; LDL-c: low-density lipoprotein cholesterol; HDL-c: high-density lipoprotein cholesterol; TG: triglycerides.

## Data Availability

The data presented in this study are available within this article or the supplementary material.

## Supplementary Materials

The following supporting information can be downloaded at: https://www.mdpi.com/article/10.3390/metabo12111121/s1, Table S1. Patients’ concomitant medication (n/N [%]) at baseline and 6, 12, and 24 months; Table S2. Univariate analysis of factors predicting an HbA1c reduction of at least 1%; Table S3. Multivariate analysis of factors predicting an HbA1c reduction of at least 1%; Table S4. Number of patients (n/%) who achieved 3% or 5% weight loss at different time points, when compared to baseline.
