# Supplementary material for: Liraglutide Effectiveness in Type 2 Diabetes: Insights from a Real-World Cohort of Portuguese Patients
Source: Metabolites. 2022 Nov 16;12(11):1121. doi: 10.3390/metabo12111121 (PMC9694138; doi:10.3390/metabo12111121)
Supplement: Supplementary file 1 [file metabolites-12-01121-s001.zip › metabolites-2000870-supplementary.pdf]

Supplementary Material

Supplementary materials to this manuscript include patient's concomitant medication at baseline and at 6, 12, and 24 months (Table S1), univariate (Table S2) and multivariate (Table S3) analyses of factors predicting an HbA1c reduction of at least 1%, and the number of patients who achieved 3% or 5% weight loss at 6, 12, and 24 months vs. baseline (Table S4).

Table S1. Patients' concomitant medication (*n*/N [%]) at baseline and at 6, 12, and 24 months.

	Baseline	6 months	12 months	24 months	*p*-values
**Anti-diabetic medication**					
Metformin	167/191 (87.4%)	156/185 (84.3%)	163/191 (85.3%)	117/139 (84.2%)	0.364 [1]<br>1.000 [2]<br>1.000 [3]
Sulphonylureas	43/191 (22.5%)	23/184 (12.5%)	27/191 (14.1%)	16/133 (12.0%)	0.001 [1]<br>0.010 [2]<br>0.026 [3]
Acarbose	19/191 (9.9%)	11/184 (6.0%)	12/191 (6.3%)	11/138 (8.0%)	0.040 [1]<br>0.137 [2]<br>0.392 [3]
Pioglitazone	6/191 (3.1%)	2/183 (1.1%)	3/191 (1.6%)	2/135 (1.5%)	0.401 [1]<br>1.000 [2]<br>0.401 [3]
DPP4i	121/191 (63.4%)	10/185 (5.4%)	10/191 (5.2%)	10/137 (7.3%)	0.000 [1]<br>0.000 [2]<br>0.000 [3]
Dapagliflozin	7/191 (3.7%)	9/185 (4.9%)	16/191 (8.4%)	22/137 (16.1%)	1.000 [1]<br>0.080 [2]<br>0.000 [3]
Insulin	108/191 (56.5%)	102/185 (55.1%)	109/191 (57.1%)	88/138 (63.8%)	1.000 [1]<br>1.000 [2]<br>0.364 [3]
**Anti-hypertensive medication**					
ACE inhibitors	44/191 (23.0%)	47/184 (25.5%)	47/191 (24.6%)	34/135 (25.2%)	1.000 [1]<br>1.000 [2]<br>1.000 [3]
ARB	98/191 (51.3%)	94/185 (50.8%)	103/191 (53.9%)	75/137 (54.7%)	1.000 [1]<br>0.683 [2]<br>0.996 [3]
Alpha/Beta-blockers	58/191 (30.4%)	56/184 (30.4%)	58/191 (30.4%)	47/135 (34.8%)	1.000 [1]<br>1.000 [2]<br>1.000 [3]
Calcium channel blockers	71/191 (37.2%)	69/185 (37.3%)	71/191 (37.2%)	56/133 (42.1%)	1.000 [1]<br>1.000 [2]<br>1.000 [3]
Renin inhibitors	1/191 (0.5%)	0/184 (0.0%)	0/191 (0.0%)	0/135 (0.0%)	NA [1]<br>NA [2]<br>NA [3]
**Diuretic medication**					
Diuretic	106/191 (55.5%)	106/185 (57.3%)	109/191 (57.1%)	79/136 (58.1%)	1.000 [1]<br>1.000 [2]<br>1.000 [3]
Aldosterone antagonists	9/191 (4.7%)	8/182 (4.4%)	8/191 (4.2%)	6/136 (4.4%)	1.000 [1]<br>1.000 [2]<br>NA [3]

**Anti-dyslipidaemia medication**					
Statins	142/191 (74.3%)	148/183 (80.9%)	150/191 (78.5%)	114/135 (84.4%)	0.010 [1]<br>0.459 [2]<br>0.026 [3]
Fibrates	36/191 (18.8%)	35/183 (19.1%)	39/191 (20.4%)	32/134 (23.9%)	1.000 [1]<br>1.000 [2]<br>0.081 [3]
Ezetimibe	18/191 (9.4%)	22/184 (12.0%)	23/191 (12.0%)	16/132 (12.1%)	0.392 [1]<br>0.802 [2]<br>0.231 [3]

ACE; angiotensin-converting enzyme; ARB, angiotensin receptor blocker; DPP4i: Dipeptidyl-peptidase 4 inhibitors. .

For multiple comparisons between baseline and the [1] 6, [2] 12, and [3] 24 months visits, the *p* value was adjusted by the Bonferroni's method.

Table S2. Univariate analysis of factors predicting an HbA1c reduction of at least 1%.

Predictors	**Beta**	**SE**	**z**	***p*-Value**	**OR**	**LL-95%CI**	**UL-95%CI**
Age	0.0275	0.0161	1.700	0.089	1.028	0.996	1.061
Disease duration	0.0166	0.0194	0.856	0.392	1.017	0.979	1.056
BMI [1]	0.0102	0.0249	0.410	0.682	1.010	0.962	1.061
Body fat mass [1]	0.0097	0.0349	0.279	0.780	1.010	0.943	1.081
Body weight [1]	0.0105	0.0152	0.693	0.488	1.011	0.981	1.041
Systolic BP [1]	0.0065	0.0079	0.821	0.411	1.007	0.991	1.022
Diastolic BP [1]	0.0109	0.0126	0.862	0.388	1.011	0.986	1.036
Total cholesterol [1]	0.0020	0.0041	0.484	0.628	1.002	0.994	1.010
LDL-c [1]	0.0027	0.0049	0.554	0.580	1.003	0.993	1.012
HDL-c [1]	-0.0023	0.0164	-0.142	0.887	0.998	0.966	1.030
TG [1]	0.0024	0.0017	1.372	0.170	1.002	0.999	1.006
HbA1c >7% [1]	3.3471	1.0257	3.263	0.001	28.420	3.807	212.193
Female sex	0.2374	0.3201	0.742	0.458	1.268	0.677	2.375
Metformin	-1.2444	0.4480	-2.778	0.005	0.288	0.120	0.693
Sulphonylureas	-0.2761	0.3818	-0.723	0.470	0.759	0.359	1.604
Acarbose	-1.5159	0.7644	-1.983	0.047	0.220	0.049	0.983
Nateglinide	NA	NA	NA	NA	NA	NA	NA
Pioglitazone	NA	NA	NA	NA	NA	NA	NA
DPP-4i	0.0746	0.3219	0.232	0.817	1.077	0.573	2.025
Dapagliflozin	-0.1904	0.8513	-0.224	0.823	0.827	0.156	4.385
Insulin	0.9093	0.3306	2.751	0.006	2.483	1.299	4.746
Diuretic	0.2523	0.3133	0.805	0.421	1.287	0.696	2.378
ACE inhibitors	0.0958	0.3639	0.263	0.792	1.101	0.539	2.246
ARB	0.1137	0.3095	0.367	0.713	1.120	0.611	2.055
Alfa/Beta blockers	0.2418	0.3315	0.729	0.466	1.274	0.665	2.439
Calcium channel blockers	0.6992	0.3168	2.207	0.027	2.012	1.082	3.744
Aldosterone antagonists	1.0084	0.6896	1.462	0.144	2.741	0.710	10.591
Renin inhibitors	NA	NA	NA	NA	NA	NA	NA
Statins	-0.0118	0.3535	-0.033	0.973	0.988	0.494	1.976
Fibrates	0.2011	0.3876	0.519	0.604	1.223	0.572	2.614
Ezetimibe	-0.2450	0.5505	-0.445	0.656	0.783	0.266	2.303
Dose adjustment <6 months	-0.3943	0.3104	-1.270	0.204	0.674	0.367	1.239

[1] Basal level. ACE; angiotensin-converting enzyme; ARB, angiotensin receptor blocker; BMI: body mass index; BP: blood pressure; DPP4i: Dipeptidyl-peptidase 4 inhibitors; HbA1c: glycated haemoglobin; HDL-c: high-density lipoprotein cholesterol; LDL-c: low-density lipoprotein cholesterol; TG: triglycerides;

Table S3. Multivariate analysis of factors predicting an HbA1c reduction of at least 1%.

Predictors	Beta	SE	z	*p*-Value	OR	LL-95%CI	UL-95%CI
HbA1c basal level >7%	3.3211	1.0294	3.226	0.001	27.691	3.682	208.237
Metformin	-1.1895	0.4862	-2.446	0.014	0.304	0.117	0.789

Table S4. Number of patients (*n*/%) who achieved 3% or 5% weight loss at different time points, when compared to baseline.

	**6 months (*n*=184)**	**12 months (*n*=191)**	**24 months (*n*=136)**
Weight reduction ≥3%	81 (44.0%)	91 (47.6%)	74 (54.4%)
Weight reduction ≥5%	45 (24.5%)	60 (31.4%)	51 (37.5%)
